# Redox Biology in Transition Periods of Dairy Cattle: Role in the Health of Periparturient and Neonatal Animals

**DOI:** 10.3390/antiox8010020

**Published:** 2019-01-13

**Authors:** Angel Abuelo, Joaquín Hernández, José L. Benedito, Cristina Castillo

**Affiliations:** 1 Department of Large Animal Clinical Sciences, College of Veterinary Medicine, Michigan State University, East Lansing, MI 48824, USA; 2 Departamento de Patoloxía Animal, Facultade de Veterinaria, Universidade de Santiago de Compostela, Lugo 27002, Spain; joaquin.hernandez@usc.es (J.H.); joseluis.benedito@usc.es (J.L.B.); cristina.castillo@usc.es (C.C.)

**Keywords:** antioxidants, calf health, dairy cattle, oxidative stress, transition period

## Abstract

Dairy cows undergo various transition periods throughout their productive life, which are associated with periods of increased metabolic and infectious disease susceptibility. Redox balance plays a key role in ensuring a satisfactory transition. Nevertheless, oxidative stress (OS), a consequence of redox imbalance, has been associated with an increased risk of disease in these animals. In the productive cycle of dairy cows, the periparturient and neonatal periods are times of increased OS and disease susceptibility. This article reviews the relationship of redox status and OS with diseases of cows and calves, and how supplementation with antioxidants can be used to prevent OS in these animals.

## 1. Introduction

Dairy cattle can succumb to illnesses at any given time. However, the majority of diseases take place around two clusters: (1) The time around calving, commonly referred to as the periparturient period, for metabolic and infectious diseases (e.g., ketosis, displaced abomasum, mastitis, metritis, etc.); and (2) the first few weeks of life, referred to as the neonatal period, for diseases of calves (e.g., diarrhea or pneumonia). These periods of increased disease susceptibility are attributed to dysfunctional immune responses in these animals. Studies performed in the last decade clearly indicate that adult dairy cows experience oxidative stress (OS) around the time of calving [1,2,3,4,5,6]. Also, some recent research has also documented that neonatal calves experience OS during the first few weeks of age [7,8,9]. OS diminishes functional capabilities of immune cell populations and increases the animals’ susceptibility to diseases [1]. Hence, the objective of this article is to review the current knowledge regarding the impact of redox biology in these periods of increased disease incidence in dairy cattle populations.

## 2. Periparturient Period

Approximately 75% of disease in adult dairy cows typically happens in the first month after calving [10], with the highest incidence of total disease (mastitis, ketosis, digestive disorders, and lameness) taking place within the first 10 days post-calving [11,12]. Notwithstanding the majority of disorders presenting after calving, the period before calving is equally relevant for their development. Most of these diseases present due to a maladaptation of the animal to the changes and demand that arise when the cows move from the pregnant and non-lactating stage to the onset of lactation [13]. For this reason, these early lactation disorders are considered “transition cow” diseases. In 1995, the transition period was defined as the period from 3 weeks pre-calving until 3 weeks post-calving [14]. The period is characterized by marked changes in the endocrine status of the animal, and a reduction in feed intake when nutrient demand for the developing fetus and the impending lactogenesis are increasing [14]. Also, dairy cows experience immune dysregulation around the time of calving, which has been linked mainly to the onset of lactation and not to parturition itself [15]. OS occurs when there is an imbalance in the redox balance that leads to cellular damage and/or dysfunction and has been proposed as the nexus between the metabolic and immune systems of the cows during this stage [3,16].

### 2.1. Factors Predisposing Cows to Oxidative Stress before Calving

As is described by several authors, animal production has increased their productiveness in the last decades. In dairy cows, Baumgard, et al. [17] pointed out that in the US, annual milk yield per cow increased over 4-fold in the last 75 years. What is the reason for this?

Historic gains in milk yield originate partly from selection and genetic improvement (50–66%) and the remainder from advances in nutrition and management. Examples explaining the historic gains include a better understanding of nutrient requirements, improvements in diet formulation and mixing, utilizing artificial insemination (AI) and applying more accurate genetic selection methods, improved milking management practices, and the effective use of herd health programs to prevent disease. Furthermore, new technologies and management tools, such as estrus synchronization, and early pregnancy detection have enhanced the production potential of dairy cows and allowed them to more closely achieve their genetic capacity [17]. As a result of these measures, dairy cows have changed their metabolism and nutrient partitioning, so that while they have been able to increase milk production, this has also led to the emergence of new metabolic diseases or even periods in which their physiology has been overwhelmed, endangering their health, as happens in the transition period.

**Figure 1:**
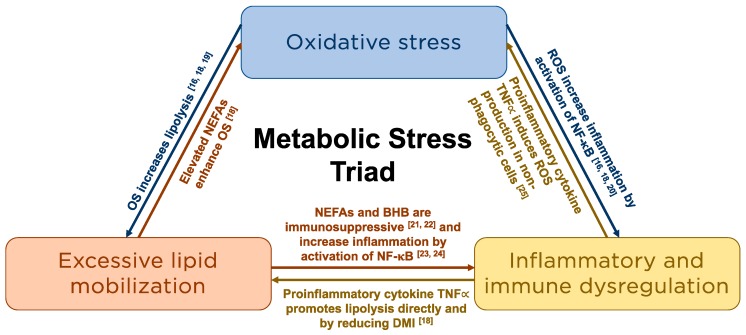
Schematic representation of the intrinsic relationships among the components of the metabolic stress triad. NEFA = non-esterified fatty acid; OS = Oxidative Stress; ROS = Reactive oxygen species; NF-κB = nuclear factor kappa-B; BHB = beta-hydroxybutyrate; TNFα = Tumor necrosis factor alpha; DMI = Dry matter intake.

Metabolic stress describes the hypermetabolic catabolic response to this disruption in physiological homeostasis [3] and is characterized by excessive lipomobilization, immune and inflammatory dysfunction, and OS (Figure 1). These three processes are intrinsically linked [16,18,19,20,21,22,23,24,25] and result in immune and metabolic derangements that are associated with an increased risk of metabolic and infectious disease during this period [16].

Currently, the transition period is one of the most stressful times in the life of dairy cows. Dairy cows go through dramatic physiological changes to prepare themselves for the onset of lactation and the climb to peak milk production. In peripartal cows, dry matter intake (DMI) decreases around parturition, whereas energy and calcium demands for lactation increase [26]. In this situation, tissues consume more oxygen through normal cellular respiration during times of increased metabolic demand in order to provide the energy needed for the onset of lactation [26,27], resulting in a negative energy balance (NEB). After calving, most cows undergo a period of NEB, in which the energy demand for milk synthesis is not covered by voluntary feed intake. To meet the increased energy demands, cows mobilize body reserves predominantly from adipose tissue. Increased lipid mobilization as a consequence of NEB may increase the generation reactive oxygen species (ROS) and reactive nitrogen species (RNS) [18,28]. An imbalance between both products coupled with the decreased intake of dietary antioxidants due to a decreased overall feed intake can lead to a pro-oxidant shift in the redox balance [1,4,29]. 

Physiologically, the cow’s body has sufficient antioxidant to counteract the production of ROS/RNS that are continuously produced during metabolism. ROS/RNS play key physiological functions, such as gene activation, cellular growth and death, or biosynthesis of prostaglandins, among others. However, the production of ROS/RNS can increase as a result of pathological conditions or the increase of physiological processes beyond the cow’s homeorhetic mechanisms. Indeed, the balance between the physiological functions of ROS/RNS and the damage they can cause is determined by the rate at which ROS/RNS are formed and removed [29]. When an imbalance occurs between the generation of ROS/RNS and the body’s antioxidative capacity, this leads to a shift in the oxidant status that can lead to oxidative/nitrosative stress when there is subsequent cellular or tissue damage or function impairment. In periparturient cows, enzymatic antioxidants, such as superoxide dismutase (SOD) and glutathione peroxidase (GSH-Px), represent the major antioxidative defense mechanisms in protecting the cells against increased ROS. Superoxide dismutase catalyzes the partitioning of the superoxide radical (O_2_^−^) into hydrogen peroxide, which is subsequently reduced to water by the GSH-Px enzyme. In cows, a high SOD activity on the day of calving has been associated with a higher degree of OS due to a lower antioxidant capacity [30]. The activity of the GSH-Px enzyme relies on the body selenium concentration. In addition to serving as a body antioxidant, selenium is required for the maintenance of other relevant biological functions, such as immune function, thyroid hormone metabolism, and reproduction [31,32].

Conversely, the proteins that afford protection against RNS under metabolic stress conditions remain unknown [28]. Several enzymes responsible for resistance to nitrosative stress have been identified through genetic studies of bacteria, including *E. coli, Salmonella enterica* serovar Typhimurium, and *Mycobacterium tuberculosis* or of the yeast, *Saccharomyces cerevisiae* [33], although a single domain haemoglobin (SdHb) and the peroxiredoxinlike protein, Prx3, seem to display a high reactivity against RNS species in infections by *Helicobacter pullorum* [34].

During pregnancy, feed is consumed, and digested products are assimilated and partitioned in a process governed by a physiological rank; meeting maintenance requirements is the top priority and secondary uses of absorbed nutrients are for productive functions, such as milk synthesis or fetal development. Further, on a short-term basis, body reserves can be replenished or mobilized to support the hierarchical goals of nutrient trafficking [17]. 

The metabolic demands imposed on the cow by colostrum production and the onset of lactation far exceed the demands of the fetus. Metabolic adaptations to lactation are initiated in late pregnancy, especially during the close-up dry period. In general, the dry period is the critical time between lactation in which a cow’s mammary gland remodels and regenerates in preparation for the ensuing lactation. During this time, high-producing dairy cows are subject to stressors, such as an abrupt cessation in milking, mammary gland discomfort, and physiological imbalances, such as hormonal dysregulation and changes in the concentration of biomarkers of nutrient utilization, OS, and inflammation [35,36]. These changes have important effects on the immune function, productivity, and health status of periparturient animals [37].

Nevertheless, these adaptations vary widely among individual cows [17]. Thus, high-yielding cows direct a greater portion of absorbed nutrients to the mammary gland for milk synthesis. In fact, previous reports point out that the estimated feed intake of the highest producing dairy cows on commercial farms is commonly incorrect in relation to concentrate feeding, and errors of more than 50% in feed intake are sometimes found [5]. Thus, the high crude protein (CP) content in the ration of pregnant cows near parturition increases urea levels, closely connected with the development of a nitrosative stress status [29], with detrimental consequences in animal health. 

On the other hand, the body condition score (BCS) determines the greater or lower predisposition to OS at calving. The study of Bernabucci et al. [30] indicates that cows with a high BCS were prone to OS status. Indeed, dairy cows with a body condition score > 3.5/5 are sensitive to OS and metabolic disorders during the transition period [38]. However, the results and recommendations made by different authors vary widely and are often contradictory. This may be due to many factors, like diet, specific requirements for certain nutrients, breed, or environmental conditions [5]. In addition to BCS at calving, the degree of BCS change around calving is also a determining factor. Various studies have documented that periparturient cows with greater BCS loss have higher non-esterified fatty acid (NEFA) concentrations and OS than those with lower BCS loss throughout the periparturient period [39,40,41].

BCS loss is associated with fat and protein breakdown. Thus, increasing the catabolic pathways in order to generate energy from lipids and amino acids. Lipid peroxidation is one of the important consequences of oxidative stress [1,4,27]. Lipid peroxidation is known to be a free radical chain reaction, which forms lipid hydroperoxides and secondary products. The latter are highly reactive and have been shown to interact with many biological components, such as proteins, aminoacids, amines, or DNA [5]. Mitochondrial DNA (mtDNA) is not protected by proteins, such as histones, so it is more susceptible to damage from OS than nuclear DNA. Damaged mtDNA can result in a decline of mtRNA transcription and lead to dysfunction of mitochondrial biogenesis [38] and uncontrolled inflammation due to dysregulated NLRP3 inflammasome activity [42]. Cellular/membrane fatty acids are highly susceptible to oxidation, which generates lipid radicals that can act on adjacent cellular lipids, creating a positive feedback loop that can result in cell damage and death [16,18,26]. Therefore, elevated serum NEFA concentrations due to excessive lipomobilization may enhance OS [18]. However, the level at which ROS result in tissue damage and increased disease susceptibility is still unknown [43]. Excessive adipose tissue mobilization is a hallmark of the transition period in dairy cows developing a metabolic stress situation that disrupts physiological homeostasis [3,16], and is related to the degree of OS experienced by the animals [39,40,41]. Indeed, decreasing OS through prepartum antioxidant supplementation resulted in improved glucose tolerance in early lactation [44], thus suggesting that when OS is reduced, nutrient utilization might also improve.

During the transition period, dairy cows also experience immune dysregulation, which increases their susceptibility to infectious and metabolic diseases. Although both endocrine and metabolic factors contribute to immune dysregulation during this period, the onset of lactation is likely the main contributing factor [15,45]. Additionally, the elevated NEFA and beta-hydroxybutyrate (BHB) concentrations from excessive lipomobilization, and hypoglycemia, as glucose is required for immune cell function, are important contributors to the periparturient immune dysregulation. Glucose is vital for proper metabolic function and immunity because it is the main metabolic fuel for many of the immune cells [46]. Low concentrations of glucose have been linked to a less effective pathogen-killing oxidative burst from polymorphonuclear neutrophils and are often seen at the same time as decreases in GSH concentrations [12], both of which impair host defenses. The stability of serum glucose concentrations in the dry period, with closely controlled nutrition, is then of particular interest [4,37] preventing reductions in maternal serum glucose due to the glucose needs of the fetus.

Nevertheless, this pro-inflammatory condition during the transition period could not be considered from a negative point of view, even if the periparturient cow has no clinical signs of disease [47,48]. In fact, inflammation aids in facilitation of parturition [49], and may also play a role in homeorhetic adaptations to the onset of lactation [50]. However, excessive and dysregulated inflammation predisposes dairy cows to metabolic and infectious diseases [18]. It is likely that excessive lipomobilization and OS contribute to excessive and dysregulated inflammatory responses during the transition period. In humans, this is associated with non-sterile inflammation due to NLRP3 inflammasome activation [51]. However, further research is needed to understand the role of the NLRP3 inflammasome in the pathophysiology of cattle diseases.

The peripartal inflammatory response is characterized by an increase in the production of positive acute phase proteins, such as haptoglobin and serum amyloid A, and a concomitant decrease in the production of negative acute phase proteins, such as albumin [47]. At the level of the liver, the well-established triggers of these responses are the proinflammatory cytokines, interleukin (IL) 6, IL-1, and tumor necrosis factor alpha (TNFα) [52]. Increased inflammatory and acute phase protein concentrations were also demonstrated in the adipose tissue of cows with higher rates of lipolysis [39,53]. Metabolic factors that contribute to immune dysregulation during this period include products of excessive lipomobilization and hypoglycemia, increasing disease susceptibility. Different dietary strategies have attempted to control lipomobilization in dairy cows. However, it is important to consider that large individual variation exists among cows on the degree of lipid mobilization [40,54], and therefore this is not only dependent on the diet fed. Cows overfed energy during the dry period have higher concentrations of NEFA and BHB compared with cows fed a controlled-energy diet prepartum. The reason for an increase in blood NEFA concentrations at the level of adipose tissue in cows overfed energy has not been fully elucidated. Initially, it was hypothesized that cows with high BHB concentrations suffered from tissue-specific decreased insulin sensitivity [55], leading to higher rates of adipose tissue mobilization in the postpartum period. Also, cows losing more body weight postpartum showed decreased adipose tissue insulin sensitivity compared to those losing less weight [39]. However, Mann et al. [35,56] considered that differences in serum concentrations of NEFA between cows overfed energy prepartum and high blood concentrations of BHB are likely due to greater NEB postpartum reflected in lower circulating concentrations of glucose and insulin and an increase in the total amount of mobilized adipose tissue mass rather than due to changes in adipose tissue insulin signaling. 

Additionally, elevated RNS are also found at this time as a consequence of high protein content in the ration. Agents that lead to protein oxidation include reagents, such as reduced transition metals, such as Fe^2+^ or Cu^+^, activated neutrophils, and by-products of lipid and free amino acid oxidation. It has been demonstrated that an increase in dietary concentrate content and a reduction in dietary neutral detergent fiber (NDF) content are associated with an increase in ruminal endotoxins, which may stimulate the production of proinflammatory cytokines, ROS, and bioactive lipids [28]. On the other hand, multiparous and primiparous animals differed significantly in serum α-tocopherol concentrations, with primiparous animals exhibiting constantly higher values than multiparous cows, associated to the different feeding strategies, such as keeping heifers on pasture for longer periods of time [57]. Indeed, pasture feeding gives higher vitamin E consumption than feeding conserved silage to housed animals [58]. 

To summarize, cows may develop metabolic stress if they fail to physiologically adapt to the profound increase in nutrient requirements associated with fetal growth, parturition, and lactogenesis during this period [16]. This status is intrinsically linked and results in immune and metabolic derangements that are associated with an increased risk of metabolic and infectious disease during the transition period [16]. In fact, 75% of disease in dairy cows occurs within the first month of lactation [10]. The influence of OS in ruminants’ health in the dry period is a relatively recent field of research [59] and the possible relationship between the ruminal activity and OS is being studied. Undesirable fluctuations in metabolites and impaired immune defense mechanisms near parturition can severely affect cow health and have residual effects on performance and longevity [60].

### 2.2. Calving: Changes Taking Place at the Onset of Lactation that Contribute to Oxidative Stress

Over the last decade, numerous studies were conducted to determine the optimal duration of the dry period length and management strategies to minimize metabolic disorders in high-producing dairy cows. Many studies reported that shortening or omitting the dry period improve postpartum energy balance because of a decrease in milk production or an increase in DMI after calving, reducing the risk of ketosis in early lactation [61,62]. However, there is still some controversy regarding the effect of omitting the dry period in the cows’ oxidant status. Mantovani et al. [62] reported no difference in concentrations of malondialdehyde, a product of lipid peroxidation, between dried-off and not dried-off cows. However, malondialdehyde has been criticized as a biomarker of OS because only a fraction of the quantified malondialdehyde is actually generated in vivo [43,63]. Conversely, cows in which the dry period was omitted showed a more pro-oxidant redox balance than cows experiencing a common 60-day long dry period when the oxidant status was assessed by measuring reactive oxygen metabolites and paraoxonase, an antioxidative enzyme [61]. Nevertheless, both studies reported that omitting the dry period resulted in an improved energy balance and no differences in the incidence of postpartum disease when compared to the traditional 55-to-60-day dry period [61,62].

Regarding the dietary changes associated with the dry period, the study of Jolicoeur et al. [64] indicates that reducing the number of diet changes before calving could facilitate ruminal adaptation to the lactation diet and improve energy balance postpartum. Indeed, when dietary changes occurring while the cow transitions from the dry to the lactating states are not done correctly, cows are at an increased risk of rumen health disorders, among which subacute rumen acidosis (SARA) is the most common. SARA usually develops in early lactating cows when there is a sudden inclusion of large amounts of concentrates in the diet to provide more energy to the animals as their energy demands to support lactation increase. However, when the dietary change is abrupt and cows develop SARA, the negative energy balance is exacerbated as it decreases intake and promotes a pro-inflammatory state. Abuelo et al. [65] showed that the concentration of lactic acid isoforms (a ruminal activity biomarker) is associated with the oxidant status of periparturient dairy cattle. Nevertheless, different roles were identified for each enantiomer, i.e., antioxidant for L-lactate and pro-oxidant for D-lactate. These findings are the first step in studying the effect of different nutrition strategies that could modulate the fermentation processes that occurs within the rumen and how these affect redox signaling and systemic OS.

Independently of a proper metabolic adaptation to the onset of lactation, there are registered high concentrations of ROS although these can be maintained by a short period of time if there are enough antioxidants that can cope efficiently with them [6]. Hence, the ratio of total oxidants to antioxidants provides a more accurate representation of the redox status of the animals. Indeed, previous contradictory findings regarding the redox status during the transition period may be a result of expressing oxidants and antioxidants separately. Hence, Celi [43] proposed using the ratio of pro- to antioxidants to monitor shifts in the redox balance of dairy cows. This was assessed by Abuelo et al. [2], who studied the redox status of dairy cows throughout the transition period using the oxidant status index (OSi), which describes the ROS to serum antioxidant capacity ratio. The authors concluded that the OSi quantifies changes in the redox balance more accurately than evaluating oxidants and antioxidants separately, as it integrates both components of redox balance.

However, ROS can also be produced because of inflammation, as they play an essential role in many inflammatory processes, such as the production of immunoregulatory factors, intra-cellular killing mechanisms, and production of lipid mediators [16]. Furthermore, ROS may also increase inflammation by activation of nuclear factor kappa-B (NF-κB) [19]. On the other hand, there are also several factors that may contribute to the reduced antioxidant capacity observed during the transition period. Firstly, increased production of ROS likely contributes to depletion of antioxidant mechanisms [66] and possibly oxidation-induced inactivation of antioxidant enzymes’ systems [67]. Secondly, there is a decrease in vitamins and minerals involved in the antioxidant defense system during this period, which is in part attributable to a loss in colostrum. Reduced hepatic function during this period may also contribute to a decreased antioxidant capacity, as the liver is responsible for the production of substances involved in the antioxidant system. Different studies suggest that a significant worsening occurs of both inflammatory and metabolic indices in transition cows after the administration of interferon-α (IFN-α) [68]). Additionally, Bradford et al. [69] showed that daily administration of tumor necrosis factor alpha (TNFα) to late-lactating cows promotes the accumulation of triglycerides in the liver. Thus, increasing the risk of fatty liver in early lactation when lipomobilization increases. Finally, the mRNA abundance of IL-6 is increased in early-lactation cows with induced ketosis [70]. This suggests that IL-6 may play a key role in the liver function dysfunction typically seen in periparturient cows. Hence, a clear correlation does exist among immune dysregulation, disease occurrence, inflammation, and metabolic stress [37].

In addition to the endogenous generation of ROS in metabolic processes, various environmental factors can also contribute to increasing ROS in dairy cows. Environmental heat stress has several detrimental effects on dairy cows’ health and wellbeing [71], including increased OS [72]. Hence, heat abatement strategies, especially during the late gestation and early lactation, are needed.

Dairy cattle are more susceptible to a variety of metabolic and infectious diseases during the transition period, probably associated directly to numerous genetic, physiological, and environmental factors that can compromise the cow’s immunological defenses [73]. The role of antioxidants in health and disease was studied extensively in animal medicine [74]. Multiple diseases most commonly occur during the periparturient period when dairy cows are known to experience OS, assuming more than half of the health expenditure of the productive life, adding other expenses for the farmer that are difficult to quantify, such as the loss of milk production, and the decrease in market value, among other consequences. 

### 2.3. Preventing Oxidative Stress: The Role of Antioxidants

In the literature, there are several strategies that have been proposed and tested as a method to avoid the development or at least minimize the development of OS status during the transition period [3,58,75]. However, it should be noted that antioxidant supplementation has shown inconsistent results on dairy cows’ health and production. Whilst most studies reported an improvement in health status or productivity, some studies have also shown no effect or even detrimental effects. The review of all the antioxidant supplementation studies is beyond the scope of this article and the readers should consult some of the relevant review articles [3,58]. Here, we will only focus on the underlying principle of most of these strategies: Increasing the animals’ antioxidant capacity so that it is better equipped to counteract the increase in free-radical production.

To decrease impaired biological function due to damage to macromolecules by ROS, living organisms have developed a complex antioxidant defense system. Endogenous antioxidants can be divided into three major groups: Enzymatic antioxidants, nonenzymatic protein antioxidants, and nonenzymatic low-molecular-weight antioxidants [74]. Of these, the nonenzymatic antioxidants are primarily responsible for the antioxidant capacity of plasma. For example, the lipid-soluble α-tocopherol (vitamin E) protects cell membranes from lipid peroxidation; ascorbic acid (vitamin C) and β-carotene are able to quench singlet oxygen and peroxyl radicals and enhance the antioxidative effect of α-tocopherol. Other vitamins, such as retinol (vitamin A), only show antioxidant activity in vitro, but not in vivo [76]. Nevertheless, the study by LeBlanc et al. [77] demonstrated that in the last week prepartum, a 100 ng/mL increase in serum retinol was associated with a 60% decrease in the risk of early lactation clinical mastitis. In addition, the authors observed significant positive associations of peripartum serum concentrations among α-tocopherol, β-carotene, and retinol.

In general terms, vitamins and certain trace minerals, such as selenium (Se), have been proven to be effective in counteracting OS and the severity of several dairy cattle diseases, such as mastitis or metritis, both through a direct antioxidant effect and by enhancing the immune response [3]. Most of the established nutritional requirements traditionally focus on deficiency situations and there is now evidence that supplementation slightly above these reported requirements can improve animal health status and performance [3], as well as the quality of the final product [78]. Nevertheless, some studies reported deleterious effects of excessive antioxidant supplementation, such as the increase of odds for mastitis due to the increased production of ROS [79,80]. Hitherto, the level to which antioxidant supplementation stops being beneficial and starts to be associated with harmful consequences remains unknown. Hence, antioxidant supplementation strategies must be implemented only to levels slightly above current recommendations unless strong scientific evidence is available to support its inclusion at a higher rate.

Different antioxidants have been considered as preventive, such as dietary conjugated linoleic acid (CLA). The proposed antioxidative effect of CLA is through its incorporation into body lipids and, thus, reducing proportions of other polyunsaturated fatty acids, in particular arachidonic acid [81]. Different CLA isomers have shown antioxidative activity in vitro and in vivo in different species, such as rats, mice, and hens, and CLA has been proposed also in dairy cows using five commercial CLA products containing approximately 12% of *cis-*9*,trans-*11 CLA and *trans-*10*,cis-*12 CLA [57]. The authors found that lipid peroxidation, in terms of thiobarbituric acid reactive substances concentration, differed significantly, with CLA-supplemented animals exhibiting lower concentrations than control animals. These results were in agreement with those found by Basirico et al. [82] in an in vitro model using bovine mammary epithelial cells, concluding that CLA-induced de novo synthesis of glutathione through enhanced γ-glutamyl cysteine ligase activity, protecting cells from oxidative damage.

Another way, exposed in the study of Osorio et al. [83], considers supplementation of basal diets with rumen-protected methionine (Met), based on the hypothesis that increasing the supply of Met could enhance liver functionality, minimizing the negative effects of fatty acid accumulation in the liver soon after parturition [84]. Indeed, supplementation with Met indicates a beneficial effect on postpartal cow performance due to a better immunometabolic status. In addition, the authors observed that cows fed Met might have relied on other antioxidant sources, such as vitamin E in the form of tocopherol or SOD, to neutralize and lower ROS concentrations arising from OS around parturition.

Clearly, nutritional management seems to be the natural way to enhance the health of dairy cows, protecting the animal from an excessive production of ROS or antioxidant loss. In the last few years, a great variety of studies have been performed in which the use of plant extracts has aimed at strengthening the use of antioxidants (especially those products derived from plant extracts due its richness in polyphenols) during the transition period in dairy cows to counteract the effects of OS, giving an added value to the final product as a source of antioxidants for the human diet, with beneficial effects in the gastrointestinal tract and other tissues [85,86]. The supplementation with polyphenols is still a developing field in dairy cattle nutrition. Recent in vitro research has also shown antioxidative effects of polyphenols in bovine cells [87,88]. Also, dietary supplementation has improved milk yield and lowered liver concentrations of triacylglycerols and cholesterol [89]. However, more research is still needed before these can be recommended for routine use on farms. Natural plant extracts as a potential source of natural antioxidants is another interesting field to explore. They have the added benefit of being perceived as a safe additive by consumers. However, the inclusion of any supplements in the diet of ruminants must be done carefully and their effects supported by evidence. For example, the administration of high dietary fat can result in adverse health events or even death [90].

The agricultural sector faces new challenges as it continues to intensify production and, therefore, supplementation with natural antioxidants constitutes a challenge for nutritionists. However, given their beneficial effects, not only in the animals’ health, but also in the quality of the final product and the absence of residual contaminants, their use is warranted. Clearly, the use of natural antioxidants, regardless of the production system, is perhaps one of the safest and most accepted nutritional strategies by consumers in line with the concepts of green economy and food fortification. However, further research is still needed to better determine the time when antioxidant supplementation is most effective as well as providing evidence-based cut-off points for antioxidant supplementation in dairy cattle.

## 3. Neonatal Period

The neonatal period of dairy calves is another time of increased disease susceptibility. High neonatal morbidity and mortality rates are consistent worldwide, making high calf loss rates an international welfare problem [91,92]. In the US dairy industry, pre-weaning morbidity and mortality rates are approximately 33% and 7–11%, respectively [93]. As newborn calves adapt to the extra-uterine life, OS may contribute to increased disease susceptibility. However, redox biology also plays an important role in several physiological processes at this stage [94]. As mentioned above for the periparturient period, it is the balance between the generation of ROS and the antioxidative capabilities of the animal that influence the development of OS and the subsequent development of systemic and localized dysfunctions. In the next sections, we discuss different stages that lead to increased oxidant status during the neonatal period, as well as the available knowledge linking OS in calves with neonatal diseases and different prevention strategies.

### 3.1. In-Utero Conditions

The negative impact of metabolic stress on the immune function, health, and production of dairy cattle during this period is well established [1,95]. Metabolic stress starts several weeks before calving [18,96] and therefore can potentially affect the fetus. There is evidence in other non-ruminant species that maternal hypothalamic-pituitary-adrenal axis stress during gestation influences fetal development and exerts carryover effects on the offspring [97,98]. Studies in humans and murine models demonstrated that suboptimal intrauterine conditions during critical periods of development leads to changes in tissue structure and function [99], which may have long-term consequences on the offspring’s physiology and disease susceptibility [97,98]. Studies in ruminants have also demonstrated that exposure to heat stress and restricted or excessive energy intake during late gestation affects the immune and metabolic function of the offspring [100,101,102,103,104]. Moreover, Monteiro et al. [105] demonstrated that the detrimental effects of in-utero exposure to heat stress on milk yield and reproductive performance extend to at least the first lactation of the offspring. Thus, prenatal conditions have the potential of significantly impacting the productivity and health status of replacement heifers.

A recent study by Ling et al. [106] compared the metabolic status and lipopolysaccharide (LPS)-induced whole blood TNFα release between calves born to cows that experienced different degrees of maternal metabolic stress during the last month of pregnancy. They found that calves born to cows with higher NEFA or OSi showed lower bodyweights at birth and throughout the study, whilst no association between any of the maternal groups and average daily gain at 4 weeks of age was identified. Serum concentrations of ROS were higher in calves exposed to higher maternal NEFA concentrations or OSi when compared to calves born to cows with lower values of these biomarkers. Calves exposed to high maternal OS also had higher circulating concentrations of haptoglobin and TNFα, indicating greater basal inflammatory responses when compared to calves born to cows with a lower OSi. In contrast, LPS-induced inflammatory responses were less robust in calves exposed to higher maternal biomarkers of inflammation or OS, suggesting compromised immune responses to microbial agonists. Collectively, their results suggest that prenatal exposure to maternal parameters of metabolic stress (altered nutrient utilization, dysregulated inflammation, and OS) may adversely impact some metabolic and inflammatory responses of the offspring that could influence disease susceptibility. Hence, the metabolic stress experienced by periparturient cows not only predisposes the cows to transition cow disorders, but also has carry-over effects on its offspring. However, further studies are still required to determine the clinical impact of these carry-over effects in the health and growth of the offspring to allow the development of adequate management practices. Nevertheless, some studies supplementing late-gestation cows with limiting amino acids or trace minerals have showed promising results in improving the immunometabolism of newborn calves [107], although the impact of such interventions in reducing calf morbidity and mortality rates remains unexplored.

The abovementioned study focused on the last month of pregnancy because this is the time when maternal periparturient immune dysfunction starts and the period with the fastest proliferation of immune cells in the bovine fetus. Nevertheless, to the best of our knowledge, it still remains unexplored whether other critical windows of maternal metabolic stress exposure that can compromise the development of the fetal immune response exist. Similarly, it still needs to be elucidated in dairy cattle if OS is a key factor in adverse pregnancy outcomes as it has been reported in humans [108,109].

### 3.2. The Oxidative Challenge of Birth

After birth, mammals are exposed for the first time to an oxygen rich environment once they start to breathe and this results in an increase in the production of ROS [110,111]. In humans, a brief oxygen exposure at birth induced a relatively long-lasting OS status [111]. Hence, birth-associated OS might have relevant impacts in calves’ cell growth, development, and death. Similar findings were identified in calves. Gaal et al. [8] found that the concentration of ROS in calves’ blood was 30% higher than in their dams shortly after birth and before colostrum ingestion. Given that pulmonary respiration and exposure to oxygen following birth are essential to maintain life, interventions to counteract birth-associated OS should focus on increasing the calves’ pool of antioxidants.

### 3.3. Oxidant Status during the Pre-weaning Period

A few studies have investigated the shifts in oxidant status during the first weeks of life in dairy calves. Gaal et al. [8] noted that the blood concentration of free radicals was lower than day 1 at days 3 and 7 of age, but increased again at 2 and 3 weeks of age. Conversely, other studies did not find an age effect in the concentration of ROS [7,9]. However, these studies used different biomarkers to assess pro-oxidant status. Nevertheless, Abuelo et al. [7] indicated a lower antioxidant status of newborn calves while they were being fed milk replacer, but these changes in antioxidant potential were not found in the study by Ranade et al. [9], where calves were fed whole milk until weaning. Milk replacers were found to have a low antioxidant capacity [112]. Thus, calves fed milk replacer might benefit from additional antioxidant supplementation.

**Figure 2:**
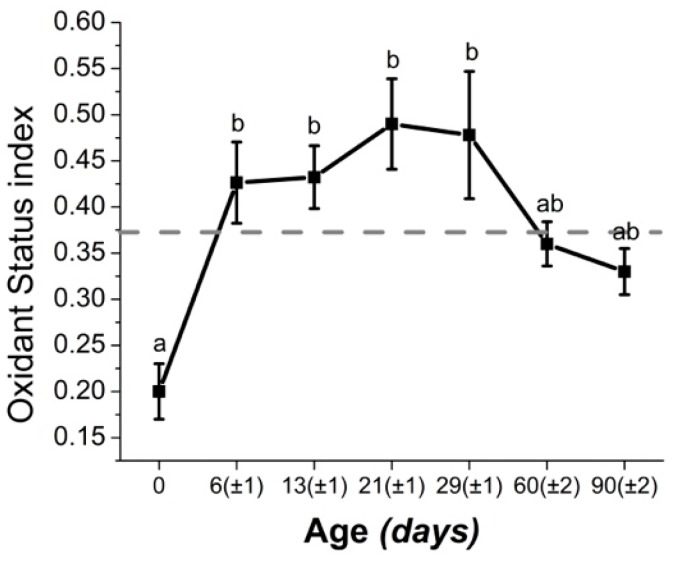
Values (mean ± SEM) of oxidant status index during the first months of life in dairy calves. The index is calculated as the ratio between reactive oxygen species and total antioxidant defenses. Hence, the higher the value of the index, the higher the imbalance of pro- to antioxidants. The dashed gray line represents the highest average index value found in periparturient dairy cattle. SEM: standard error of the mean. a, b Means with different letters are statistically different (P < 0.05) Figure adapted from [7].

Of particular interest is to note that biomarkers of the oxidant status in calves were higher than those of periparturient cattle (Figure 2) [7,8]. Hence, OS might play a very significant role in neonatal calf health. Indeed, OS is known to play a key role in the initiation and maintenance of important calf diseases, such as diarrhea or pneumonia [75,113]. The readers are encouraged to consult the review by Celi [59] for a detailed description of the role of OS in disorders of ruminants.

### 3.4. Preventive Measures and Future Research

As for periparturient cattle, several strategies exist to decrease the risk of OS in neonatal calves. Below, we summarize some of the most common ones, identifying some of the gaps in knowledge that are still present.

#### 3.4.1. Maternal Supplementation of Antioxidants

Supplementation of antioxidants during the dry period slightly above National Research Council (NRC) [114] requirements has shown beneficial effects for cow health and productivity [3]. However, given the carry-over effect of maternal OS on neonatal metabolic and immune function, this practice can also have beneficial effects in the offspring. However, research proving the effects of maternal antioxidant supplementation on calf morbidity and mortality rates is, to the best of our knowledge, non-existing to date.

In humans, antenatal supplementation of antioxidant vitamins and minerals has long been a recommended practice to reduce OS at delivery [115,116]. Some studies in cattle have also shown that dry-period antioxidant supplementation enhances the antioxidative profile of newborn calves [117,118,119]. Nevertheless, various factors limit this route in cattle: (1) The epitheliochorial nature of the ruminants’ placenta limits the types of antioxidants that can be transmitted transplacentally, (2) dry dairy cattle are usually already supplemented with considerable amounts of some antioxidants (e.g., selenium close to the US legal limit of 0.3 ppm) for the prevention of transition diseases, and (3) excessive antioxidant supplementation can have downstream effects in the health of dairy cattle [3,79,80] and has been linked with stillbirths in humans [120]. Hence, dry cows should not receive antioxidants in amounts significantly exceeding the NRC [114] requirements.

#### 3.4.2. Colostrum: A Source of Antioxidants, but also Pro-oxidants

The importance of colostrum ingestion to the health of the neonatal calf has been well-known for several decades [121]. However, this has been primarily attributed to the acquisition of passive immunity (immunoglobulins) to fight infectious diseases, with calves experiencing failure of passive transfer showing decreased survival rates on farms compared to those with adequate blood immunoglobulin concentrations [122]. In addition to immunoglobulins, colostrum is also rich in other beneficial substances, such as immune cells, growth factors, cytokines, etc. [123]. Given that colostrum is the first meal that a calf should receive shortly after birth, its antioxidant content is important to offset the birth-associated OS. However, compared to normal milk, colostrum has the same amount of oxidants, but less antioxidants, with the concentration of the latter increasing progressively from the first milked colostrum onwards [124,125]. Hence, colostrum provides antioxidants to calves, but is also a source of pro-oxidants. Nevertheless, newborn calves seem to be able to counter effectively the birth-associated OS [8], with calves showing a gradual decrease in oxidant status biomarkers [126,127,128]. Indeed, Abuelo et al. [7] found that 2 h after colostrum ingestion, calves showed the lowest OSi values of the first months of life. To the best of our knowledge, however, no study has hitherto compared the redox balance between calves that ingest colostrum shortly after birth with those experiencing delayed colostrum ingestion. Hence, it remains unexplored whether this gradual decline in OS following birth is due to the transfer of antioxidants via colostrum, the activation of antioxidative pathways in the calves, or a combination of both.

In addition, colostrum redox balance seems to play a role in immunoglobulin absorption. Selenium supplementation of colostrum increases immunoglobulin absorption [127], and the colostrum redox profile was significantly associated with calves’ serum immunoglobulin concentrations [7]. However, none of these studies demonstrated which mechanisms might be implicated and therefore further research is needed. Also, a negative association between colostrum immunoglobulin content and antioxidant capacity has been reported [7]. The authors attributed this finding to a consumption of antioxidants in protecting from peroxidation the highly-susceptible immunoglobulins during the colostrogenesis process. Therefore, supplementation of colostrum with antioxidants seems to have additional benefits to the calf beyond counteracting the birth-associated OS.

Also, there is now a plethora of research indicating the long-term impact of early-life events and management in the calves’ productive life once they reach maturity [128]. Hence, studies investigating the long-term implications of supplementation of colostrum with antioxidants in the animals’ health and productivity are also required.

#### 3.4.3. Supplementation of Calves with Antioxidants

Other ways of increasing the antioxidant potential of calves are the parenteral or dietary administration of vitamins and trace elements. This is a routine management practice in many farms within the first days of life. It has been well-stablished that vitamin supplementation of dairy calves can increase their performance, metabolism, and immune system [129,130,131,132]. Parenteral trace mineral supplementation (zinc, selenium, manganese, and copper) at 3 and 30 days of life resulted in increased neutrophil function and GSH-Px activity and decreased incidence of health disorders when compared to the control group [133]. Also, trace mineral supplementation concurrent with a polyvalent viral vaccine administration at 30 days of age resulted in improved cell-mediated immune responses [134]. However, whether this observed increase improves the vaccine’s protection against infection remains unknown.

It is important to note, however, that the NRC [114] requirements were initially developed to prevent deficiencies and there are no clear guidelines of the levels of antioxidant supplementation for optimized performance. Considering that it is likely that, as happens in adult cows, excessive antioxidant supplementation can have detrimental effects on calf health and performance, caution must be exerted when supplementing antioxidants above levels deemed safe by the scientific literature. Indeed, there are reports of toxicosis due to excessive supplementation [135]. This might be even more relevant for those antioxidants, such as selenium, that can be transferred via the placenta when the dams are also supplemented.

## 4. Conclusions

To sum up, redox balance is essential for several biological processes of dairy cows and calves. However, when an imbalance exists between the production of pro-oxidants and the animals’ antioxidant abilities, OS can develop, and this has been associated with immune and metabolic dysfunction. Also, in pregnant animals, the degree of OS experienced not only puts the dams at risk of subsequent diseases during the onset of lactation, but also has an impact on the offspring. However, antioxidant therapy can protect against OS-conditions, and several methods for the delivery of antioxidants are routinely used in dairy farms. Nevertheless, the findings have been inconsistent at times, with some studies not showing an effect. Hence, more research is still needed to provide evidence-based guidance on the levels and timing of supplementation that provide an effective improvement of the animals’ health status.
